# The role of extracellular vesicles in intercellular communication within the oral cavity

**DOI:** 10.1080/20002297.2026.2673760

**Published:** 2026-05-22

**Authors:** Abdelhabib Semlali, Mohammed Al-zharani, Jassem Al-Ansari

**Affiliations:** a Groupe de recherche en écologie buccale, Faculté de médecine dentaire, Université Laval, Québec, QC, Canada; b Biology Department, College of Science, Imam Mohammad Ibn Saud Islamic University (IMSIU), Riyadh, Saudi Arabia; c College of Health Sciences, Kuwait

**Keywords:** Extracellular vesicles, EVs biogenesis, EVs biomarkers, therapeutic applications, oral diseases

## Abstract

**Background:**

Extracellular vesicles (EVs), including exosomes, microvesicles, and apoptotic bodies, are nanoscale membrane-bound structures that mediate intercellular communication through the transfer of proteins, lipids, and nucleic acids. In the oral cavity, EVs are present in saliva and gingival crevicular fluid, where they contribute to immune regulation, epithelial barrier integrity, and host–microbiome interactions.

**Objective:**

This review aimed to critically summarize current knowledge regarding EV biogenesis, molecular composition, cellular and microbial sources, and their roles in oral homeostasis and disease pathogenesis.

**Results:**

Current evidence demonstrates that EVs derived from host cells and oral microorganisms actively regulate inflammatory responses, biofilm dynamics, tissue regeneration, and microbial colonization. Dysregulation of EV-mediated communication has been implicated in the development and progression of periodontal disease, oral candidiasis, and oral squamous cell carcinoma (OSCC). In addition, salivary EVs have emerged as promising non-invasive biomarkers and potential therapeutic tools in oral medicine. However, important methodological limitations remain, particularly regarding EV isolation, characterization, and standardization.

**Conclusions:**

EVs represent central mediators of communication within the oral microenvironment and play dual roles in maintaining oral homeostasis and promoting disease progression. Further standardized and clinically relevant studies are required to better define EV biology and facilitate the translation of EV-based diagnostic and therapeutic strategies into clinical oral medicine.

## Introduction

The oral cavity constitutes one of the most dynamic and complex biological environment**s** in the human body, functioning as the primary interface between the external environment and the gastrointestinal and respiratory tracts [1–3]. Its structural organisation comprises multiple specialised tissue, including keratinised and non-keratinised stratified squamous epithelium, connective tissue rich in fibroblasts and extracellular matrix components, and specialised glandular structures responsible for saliva secretion [4]. Beyond its structural functions, the oral mucosa serves as both a physical and immunological barrier. The junctional epithelium is particularly permeable, allowing rapid leucocyte migration and immune surveillance [5,6]. The oral mucosa hosts an extensive immune network composed of resident and infiltrating immune cells including neutrophils, macrophages, dendritic cells, Langerhans cells, and lymphocytes, which together regulate immune tolerance toward commensal microorganisms while maintaining the ability to respond rapidly to pathogens [7,8]. This environment supports a highly diverse oral microbiome, considered the second most complex microbial community after the gut, comprising more than 700 bacterial species as well as fungi (e.g. *Candida albicans*, *Candida glabrata*), viruses (e.g. Human herpesvirus 1), archaea (e.g. *Methanobrevibacter oralis*) and protozoa (e.g. *Entamoeba gingivalis*) [2,9].

These microorganisms colonise distinct ecological niches such as dental plaque, the tongue dorsum, gingival crevice, saliva, and mucosal surfaces, each characterised by unique conditions of pH, oxygen tension, nutrient availability, and mechanical forces [10]. Such microhabitats promote the development of structured biofilms, particularly dental plaque, which plays a pivotal role in oral diseases [11]. Under physiological conditions, host–microbe interactions maintain homoeostasis, with commensal microorganisms contributing to colonisation resistance, immune maturation, and metabolic functions such as nitrate reduction [12,13]. However, disruption of this ecological balance leads to dysbiosis, allowing pathogenic species such as *Streptococcus mutans*, *Porphyromonas gingivalis*, and *C. albicans* to dominate and trigger inflammatory responses and tissue damage [14,15].

Within this complex ecosystem, extracellular vesicles (EVs) have emerged as critical mediators of intercellular communication. EVs are membrane-bound nanoparticles released by virtually all cell types and capable of transporting bioactive cargo such as proteins, lipids, nucleic acids, and metabolites between cells [16,17]. Through the transfer of this molecular cargo, EVs regulate in numerous physiological and pathological processes such as immune modulation, inflammation, tissue repair, and cancer progression [18,19].

EVs are commonly categorised into three major classes according to their biogenesis: exosomes, microvesicles, and apoptotic bodies [20–22].

Exosomes are small EVs typically ranging from 30–150 nm that originate from the endosomal pathway, where inward budding of endosomal membranes forms intraluminal vesicles within multivesicular bodies (MVBs) [21,23]. Fusion of MVBs with the plasma membrane subsequently releases exosomes into the extracellular environment [24,25]. These vesicles are enriched in characteristic markers including tetraspanins (CD9, CD63, CD81), heat-shock proteins, and ESCRT-associated components reflecting their endosomal origin [26,27].

Microvesicles, also referred to as ectosomes or shedding vesicles, are larger structures (100–1000 nm) generated by direct outward budding and fission of the plasma membrane [28,29]. Their formation involves cytoskeletal remodelling, phospholipid redistribution, and calcium-dependent signalling processes [30]. As a result, microvesicles frequently contain plasma membrane proteins, integrins, and cytosolic components that mirror the activation state of their parental cell [31,32].

**Figure 1.**
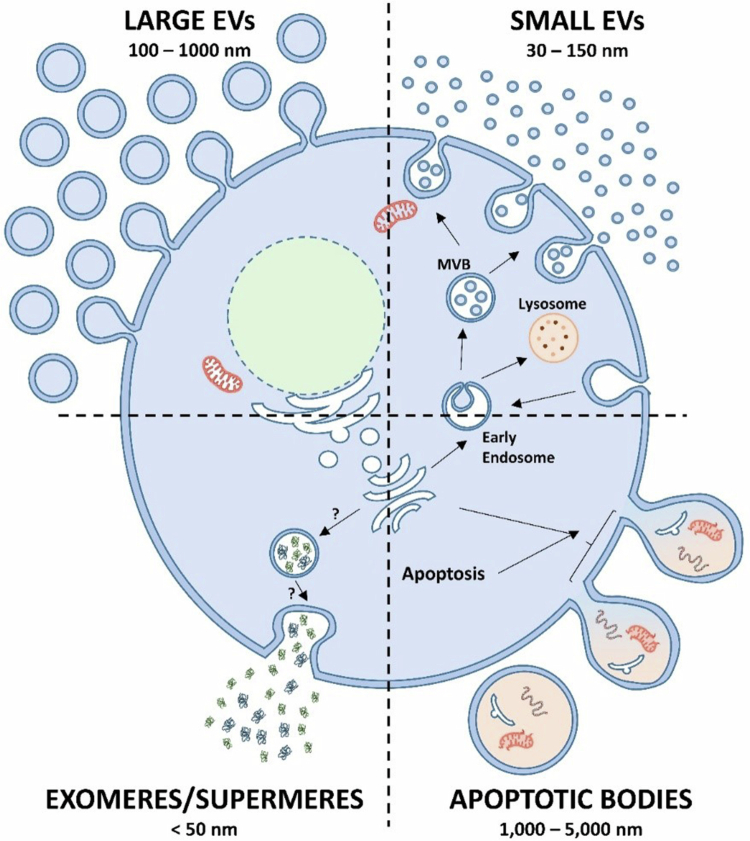
Biogenesis pathways and size ranges of extracellular vesicles (EVs). This schematic illustrates the major classes of EVs and their cellular origins. Small EVs (30–150 nm), primarily exosomes, originate from the endosomal pathway. Early endosomes mature into multivesicular bodies (MVBs) containing intraluminal vesicles (ILVs). Upon fusion of MVBs with the plasma membrane, ILVs are released as exosomes. Alternatively, MVBs may fuse with lysosomes for degradation. Large EVs (100–1000 nm), often referred to as microvesicles or ectosomes, are generated through direct outward budding and fission of the plasma membrane, a process dependent on cytoskeletal remodelling and phospholipid redistribution. Apoptotic bodies (1000–5000 nm) arise during the late stages of programmed cell death as the cell fragments into membrane-enclosed vesicles containing nuclear components, organelles, and cytoplasmic material. The figure also depicts exomeres/supermeres (<50 nm), nanoscale particles known to carry distinct metabolic and signalling cargo (BioRender Software and ameliorate by chatgpt).

Apoptotic bodies represent the largest subtype of EVs (500–5000 nm), and arise during programmed cell death as the cell fragments into membrane-enclosed vesicles [33] containing nuclear fragments, organelles, and cytoplasmic materials [34]. Unlike exosomes and microvesicles, apoptotic bodies primarily reflect cellular disassembly rather than active communication mechanisms [20] (Figure 1).

Because EV subtypes exhibit overlapping size ranges, biogenesis pathways, and molecular markers, their precise classification remains challenging. Recent consensus guidelines therefore recommend categorising EVs based on physical characteristics, biochemical composition, and cellular origin rather than strict terminology [35,36]. This evolving nomenclature highlights the complexity of EV biology and emphasises the need for standardised methodologies for EV isolation and characterisation.

Within the oral cavity, EVs mediate complex communication networks between host cells, immune components, and resident microbiota [7,37,38]. Both host-derived and microorganism-derived EVs transport biologically active cargo including proteins, lipids, RNA species (mRNA, miRNA), DNA fragments, and metabolites, enabling the transfer of functional signals across local and systemic environments [39–41]. Locally, EVs regulate epithelial barrier integrity, immune responses, microbial colonisation, and biofilm dynamics [42]. For example, epithelial cell EVs can modulate neutrophil recruitment and cytokine production, supporting mucosal defence mechanisms [43], while immune cell-derived EVs influence antigen presentation and inflammatory signalling [44].

**Figure 2.**
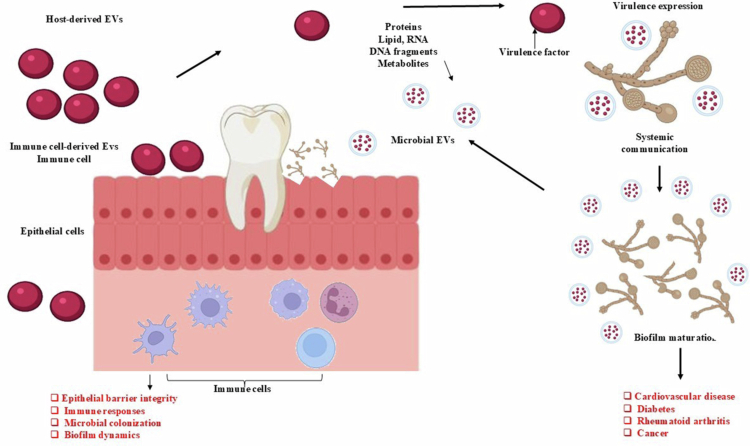
Interplay Between Host-Derived and Microbial Extracellular Vesicles (EVs) in Oral Mucosal Homoeostasis, Microbial Virulence, and Systemic Disease. On the left, host-derived EVs including epithelial cell-derived EVs and immune cell-derived EVs play a role in the maintenance of oral mucosal homoeostasis by regulating epithelial barrier integrity, modulating immune responses, influencing microbial colonisation, and shaping biofilm dynamics. In the centre, microbial EVs containing proteins, lipids, RNA, DNA fragments, and metabolites interact directly with epithelial and immune cells, delivering virulence-associated cargo and modulating host pathways. On the right, microbial EVs promote virulence expression, enhance biofilm maturation, and mediate systemic communication. Through circulation or translocation, these EVs may contribute to the development or exacerbation of systemic inflammatory conditions, including cardiovascular diseases, diabetes, rheumatoid arthritis, and cancer (BioRender Software).

Microbial EVs also play an important role in oral ecological interactions. Gram-negative bacteria such as *Porphyromonas gingivalis* release outer membrane vesicles enriched with proteases and lipopolysaccharides that modulate host immune responses and contribute to periodontal tissue destruction [45–48]. Similarly, fungal EVs produced by *Candida albicans* contain virulence-associated enzymes and cell wall components that enhance adhesion, invasion, and immune evasion [49] (Figure 2).

Importantly, EVs originating from oral tissues or microorganisms can enter the systemic circulation through gingival crevicular fluid, periodontal vascular permeability, or swallowing, enabling communication between the oral cavity and distant organs [50,51]. Importantly, EVs originating from oral tissues or microorganisms can enter the systemic circulation through gingival crevicular fluid, periodontal vascular permeability, or swallowing, enabling communication between the oral cavity and distant organs [50,51]. Increasing evidence suggests that EV-mediated dissemination of inflammatory mediators, microbial components, and genetic material may contribute to systemic diseases including cardiovascular disorders, diabetes, rheumatoid arthritis, and cancer progression [52–56].

Collectively, EVs function as critical mediators linking oral microbiology, immune regulation, and systemic health. Understanding EV-mediated communication within the oral ecosystem may therefore provide important insights into disease mechanisms and support the development of novel diagnostic and therapeutic strategies in oral medicine [57].

## Literature search strategy

A structured literature search was conducted to identify relevant studies addressing extracellular vesicles (EVs) in the oral cavity. The databases PubMed, Scopus, and Web of Science were searched for articles published up to [February 2026]. Keywords included combinations of *“extracellular vesicles”, “exosomes”, “microvesicles”, “outer membrane vesicles”, “oral cavity”, “oral microbiome”, “periodontitis”, “oral cancer”, “Candida”, “biofilm”, and “host–microbe interactions”*. Priority was given to recent peer-reviewed studies and seminal publications describing EV biogenesis, molecular composition, host–microbiome communication, and their roles in oral health and disease. Both experimental studies (in vitro and in vivo) and clinical studies were considered to provide a comprehensive overview of current knowledge.

This article was designed as a critical narrative review, aiming to synthesise and critically evaluate existing evidence rather than perform a formal systematic review. Particular attention was given to methodological heterogeneity in EVs isolation and characterisation, limitations of current experimental models, and remaining knowledge gaps in oral EVs research.

## Biogenesis and composition of oral EVs

The biogenesis of EVs within the oral cavity reflects the diversity of local cellular and microbial sources, resulting in heterogeneous vesicle populations with distinct structural and molecular characteristics [58]. Exosomes originate from the endosomal pathway, beginning with the inward budding of the early endosomal membrane to form intraluminal vesicles (ILVs) within multivesicular bodies (MVBs) [20,24]. The maturation and sorting of cargo into ILVs are regulated by the endosomal sorting complex required for transport (ESCRT) machinery, including TSG101 and ALIX, as well as ESCRT-independent mechanisms involving ceramide and tetraspanin-enriched microdomains [59,60]. Following MVB fusion with the plasma membrane, ILVs are released as exosomes into the extracellular milieu [61]. In the oral cavity, epithelial cell-derived exosomes contribute to mucosal barrier regulation and immune signalling, while immune cell exosomes modulate inflammatory responses and antigen presentation [62,63].

In contrast, microvesicles are produced through direct outward budding and fission of the plasma membrane, driven by cytoskeletal remodelling, calcium influx, and phospholipid redistribution [64,65]. During this process, exposure of phosphatidylserine on the outer leaflet and actin–myosin contraction facilitates membrane scission, releasing microvesicles that carry surface proteins, integrins, and cytosolic components reflective of their parental cell activation state [66,67]. Within oral biofilms, bacterial and fungal cells such as *P. gingivalis* and *C. albicans* generate vesicles through membrane blebbing, contributing to virulence factor dissemination and interspecies communication [68,69].

Apoptotic bodies form during late stages of programmed cell death as dying cells undergo fragmentation into membrane-bound vesicles containing nuclear fragments, organelles, and cytoplasmic material [70,71]. In periodontal inflammation, elevated epithelial turnover and immune cell apoptosis increase apoptotic body release, facilitating clearance by phagocytes and promoting immune tolerance [72].

**Figure 3.**
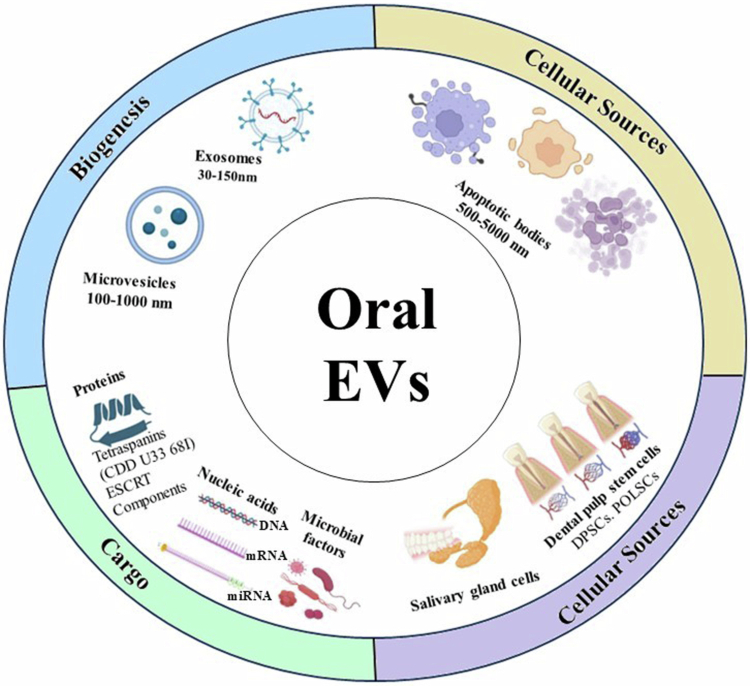
A circular illustration showing how the biogenesis pathways, molecular cargo, and diverse cellular origins combine to generate a heterogeneous EVs population that orchestrates oral homoeostasis, host–microbe interactions, immune responses, and systemic communication. Biogenesis: Exosomes (30–150 nm) are formed by inward budding of endosomal membranes and released following fusion of multivesicular bodies with the plasma membrane, regulated by ESCRT proteins (TSG101, ALIX), ceramide, and tetraspanins. Microvesicles (100–1000 nm) arise from outward budding of the plasma membrane, while apoptotic bodies (500–5000 nm) derive from membrane blebbing during late apoptosis. Cargo: Oral EVs contain proteins (tetraspanins CD9, CD63, CD81; HSP70/90; ESCRT components), lipids (ceramide, sphingomyelin, phosphatidylserine), nucleic acids (mRNA, miRNA, lncRNA, DNA fragments, mtDNA), and microbial factors (LPS, gingipains, adhesins, glucan-modifying enzymes), enabling potent host–microbe communication. Cellular sources: EVs originate from multiple oral cell types including epithelial cells (keratinocytes), immune cells (neutrophils, macrophages), dental pulp stem cells (DPSCs, PDLSCs), and salivary gland cells. Epithelial EVs regulate mucosal integrity and microbial colonisation; immune cell EVs carry antimicrobial mediators and inflammatory signals; stem cell EVs promote dentin–pulp regeneration and periodontal repair; salivary EVs serve as stable carriers of biomarkers for systemic and oral diseases (BioRender Software and ameliorate by chatgpt).

The molecular composition of oral EVs is highly diverse and reflects both their biogenesis pathway and cellular origin. Exosomes are enriched in tetraspanins (CD9, CD63, CD81), ESCRT components (TSG101, ALIX), heat shock proteins (HSP70, HSP90), and specific lipids such as cholesterol, sphingomyelin, and ceramide [73]. Microvesicles typically contain plasma membrane proteins, integrins, cytoskeletal proteins (actin, tubulin), and signalling molecules [28]. Both exosomes and microvesicles transport nucleic acids, including mRNA, microRNA, long non-coding RNA, DNA fragments, and mitochondrial DNA, enabling gene regulation and host–microbe communication [74]. Microbial EVs frequently carry LPS, gingipains, adhesins, glucan-modifying enzymes, and *β*-glucans, enhancing pathogenic potential in oral dysbiosis [75] (Figure 3).

The complex protein, lipid, and nucleic acid cargo of oral EVs positions them as potent mediators of local and systemic signalling, with substantial implications for disease development and biomarker discovery. Their heterogeneous composition also underscores the need for standardised isolation and characterisation methods, as highlighted in recent EVs consensus guidelines [76–78].

## Cellular sources of EVs in the oral cavity

The oral cavity contains multiple cellular sources capable of producing EVs (EVs), contributing to the complexity of local and systemic communication pathways. Among these, oral epithelial cells, immune cells, mesenchymal stem cell populations, and salivary gland cells represent the most abundant and biologically relEVsant producers of EVs within this environment [57,58,79].

### Epithelial cells

Including oral keratinocytes, continuously release EVs as part of their barrier maintenance and immune regulatory functions [80,81]. These vesicles carry tetraspanins (CD9, CD63), cytokines, antimicrobial peptides, and regulatory microRNAs that modulate neutrophil chemotaxis, epithelial tight junctions, and microbial colonisation [37,82,83]. Keratinocyte-derived EVs can influence biofilm composition and host inflammatory responses, contributing to mucosal homoeostasis or, under dysbiotic conditions, promoting inflammatory signalling associated with periodontitis and oral mucosal pathology [56,84].

### Immune cells

Represent another major EVs source in the oral cavity, particularly neutrophils and macrophages, which are highly active in the gingival crevice and inflamed periodontal tissues [85,86]. Neutrophil-derived EVs transport antimicrobial proteins such as defensins and myeloperoxidase, supporting innate defence mechanisms against bacterial and fungal pathogens [87]. Macrophage-derived EVs contain cytokines, MHC class II molecules, and miRNAs that regulate antigen presentation and inflammatory signalling, shaping local immune responses and contributing to tissue destruction or repair depending on activation state [88,89].

### Dental pulp stem cells

EVs production is also prominent in oral-derived stem cell populations, including dental pulp stem cells (DPSCs) and periodontal ligament stem cells (PDLSCs), which release vesicles enriched in growth factors, angiogenic mediators, and regenerative microRNAs [90,91]. These stem cell EVs have demonstrated the capacity to promote dentin–pulp regeneration, periodontal tissue repair, osteogenesis, and immunomodulation, positioning them as promising therapeutic tools in regenerative dentistry [91].

### Salivary gland cells

Secrete large quantities of EVs into saliva, providing a major route for vesicle dissemination within the oral cavity and beyond [92,93]. Salivary EVs contain proteins, lipids, and nucleic acids reflective of glandular physiology and systemic health [93]. Their stability in saliva and accessibility make them attractive candidates for non-invasive biomarker discovery in oral cancer, periodontal disease, and systemic conditions such as diabetes and cardiovascular disease [94].

Together, these diverse cellular sources generate a rich and functionally heterogeneous EVs population that orchestrates host–microbe interactions, immune regulation, tissue maintenance, and systemic signalling within the oral ecosystem [42,95]. Understanding the contribution of each EVS-producing cell type is essential for elucidating oral disease mechanisms and developing EVs-based diagnostic and therapeutic strategies.

### Oral microbiota as a source of extracellular vesicles

In addition to host-derived vesicles, the oral microbiota including bacteria and fungi constitutes a major source of extracellular vesicles that significantly influence host physiology and mucosal biology. Gram-negative bacteria release outer membrane vesicles (OMVs), while Gram-positive bacteria and fungi secrete membrane vesicles (MVs) enriched with lipids, proteins, nucleic acids, and virulence factors [75,96].

#### Bacterial EVs

Pathogenic and commensal bacteria in the oral cavity generate EVs that can diffuse through mucus and epithelial layers, delivering molecular cargo to host cells. *P. gingivalis*, *Fusobacterium nucleatum*, *Aggregatibacter actinomycetemcomitans*, and *Treponema denticola* all produce OMVs containing lipopolysaccharides (LPS), gingipains, peptidoglycans, adhesins, and outer membrane proteins that modulate immune responses and promote colonisation [56,68,75]. These bacterial EVs participate in inter-species communication, biofilm organisation, epithelial barrier disruption, and immune evasion [97].

#### Fungal EVs

*Candida albicans* also produces extracellular vesicles carrying *β*-glucans, mannoproteins, proteases, and secreted enzymes that contribute to adhesion, hyphal formation, epithelial invasion, and cross-kingdom interactions with bacteria [98]. Fungal EVs can modulate macrophage activation and influence epithelial cytokine profiles, further impacting host–microbiome dynamics [98].

Microbiota-derived EVs act as key mediators of host–microbe communication, influencing:

- **Mucosal immunity**, by activating or suppressing innate immune pathways [68,79].
- **Epithelial homoeostasis**, through modulation of tight junctions and stress responses [56,87].
- **Biofilm dynamics and microbial ecology**, by transporting quorum-sensing molecules and virulence determinants [75,99].
- **Disease progression**, including periodontitis, candidiasis, and OSCC-associated inflammation [48,100,101].

As such, the oral microbiota represents an essential and highly active EVs-producing system that contributes substantially to both oral health and disease.

## The role of EVs in oral disease pathogenesis

Extracellular vesicles (EVs) contribute significantly to the development and progression of major oral diseases by modulating inflammatory pathways, altering epithelial and immune cell behaviour, and reshaping host–microbiome interactions. ensuring the stability of the oral ecosystem [102]. Dysregulated EVs signalling whether originating from host tissues, immune cells, or the oral microbiota can shift the local environment from homoeostasis to pathological inflammation, tissue breakdown, or malignant transformation. This section summarises the role of EVs in periodontal disease, oral candidiasis, and oral squamous cell carcinoma (OSCC), the three major EVs-associated oral pathologies.

### EVs in tissue regeneration and repair

EVs derived from oral mesenchymal stem cells, particularly dental pulp stem cells (DPSCs) and periodontal ligament stem cells (PDLSCs), exert potent regenerative effects. These vesicles deliver pro-repair cargo, including growth factors, angiogenic mediators (VEGF-related signals), osteogenic microRNAs (miR-21, miR-135b), and anti-inflammatory molecules that promote tissue regeneration [90,91]. These regenerative properties position stem cell–derived EVs as promising cell-free therapeutic tools in regenerative dentistry and periodontal tissue engineering [91]. Stem-cell EVs play a role in:

- **Dentin–pulp regeneration:** Stimulating odontoblast differentiation, dentin bridge formation, and pulp vascularisation.
- **Periodontal tissue repair:** Enhancing fibroblast proliferation, extracellular matrix deposition, and periodontal ligament regeneration.
- **Alveolar bone formation:** Promoting osteoblast differentiation and reducing osteoclastogenesis.
- **Immunomodulation:** Suppressing excessive inflammatory responses that impede healing.

These regenerative properties position MSC-derived EVs as promising cell-free therapeutic tools for regenerative endodontics and the reconstruction of periodontal tissues [103].

### EVs in immune modulation and mucosal balance

EVs produced by oral keratinocytes and immune cells are central to maintaining immune equilibrium within the mucosa.

**Epithelial EVs regulate** [104]:

- Tight-junction protein expression
- Epithelial barrier integrity
- Neutrophil recruitment through chemokine signalling
- Local antimicrobial defences (e.g. *β*-defensins, LL-37)

**Immune cell EVs regulate:**

- Macrophage polarisation (M1 pro-inflammatory → M2 reparative phenotypes)
- Cytokine secretion and inflammatory cascades
- Antigen presentation and adaptive immune activation

Together, these vesicles help maintain a balanced immune state that can respond to pathogens without causing tissue damage. Under dysbiosis conditions, however, host EV composition shifts toward a pro-inflammatory profile, contributing to chronic periodontal inflammation and mucosal degradation [105,106].

### EVs in host–microbiome communication

A central function of oral EVs is the facilitation of bidirectional communication between host tissues and the resident microbiota [42].

#### Host-derived EVs influence microbiota by:

- Regulating microbial colonisation patterns [98,99].
- Altering biofilm structure and density [56].
- Delivering antimicrobial peptides and regulatory microRNAs to modulate microbial gene expression [100].

#### Microbiota-derived EVs influence host tissues by:

- Delivering virulence factors (e.g. gingipains, LPS, adhesins) that modulate immune responses [48].
- Inducing oxidative stress and inflammatory pathways [107].
- Enhancing cross-kingdom interactions (e.g. *C. albicans* with bacteria) [108,109].

These interactions illustrate the finely tuned ecological balance between the host and microbiome, which is critical for oral health.

### Dysregulation of EV-mediated communication in disease

Disruptions in EV composition, release, or uptake contribute to multiple oral diseases:

#### Periodontal disease

In periodontal regeneration, EVs contribute to periodontal ligament cell proliferation, osteogenic differentiation, and alveolar bone regeneration². Periodontal ligament stem cell–derived EVs enhance the expression of osteogenic transcription factors such as RUNX2 and bone matrix proteins including osteocalcin (OCN) [106], promoting cementum and bone formation while modulating inflammatory responses within periodontal tissues. These regenerative properties highlight the therapeutic potential of EVs in treating periodontitis-associated tissue destruction, particularly when combined with biomaterial scaffolds or targeted delivery systems.

EVs also play a significant role in cartilage regeneration, particularly in the context of temporomandibular joint osteoarthritis (TMJOA). EVs derived from mesenchymal sources can regulate chondrocyte activity, suppressing pro-inflammatory cytokines such as IL-1β and TNF-*α* while increasing anti-inflammatory mediators including IL-10 [105]. Additionally, EVs promote extracellular matrix synthesis and inhibit cartilage degradation, offering novel strategies for managing TMJ degenerative disorders through minimally invasive, biologically driven therapies [103].

In contrast to their regenerative roles, EVs also contribute to oral cancer progression by promoting angiogenesis, tumour growth, and metastasis⁴. Tumour-derived EVs can enhance vascular endothelial growth factor (VEGF) expression and stimulate endothelial cell migration, thereby supporting tumour vascularisation and invasive potential [109]. These vesicles also carry oncogenic microRNAs and proteases that remodel the tumour microenvironment, suppress immune surveillance, and facilitate metastatic dissemination [37]. As such, EVs represent both therapeutic targets and potential biomarkers for oral squamous cell carcinoma, with implications for diagnosis and treatment.

Collectively, these findings underscore the dual roles of EVs as mediators of regeneration and disease within oral tissues. Continued investigation into their mechanisms and therapeutic applications may enable the development of targeted EVs-based regenerative therapies and cancer interventions, advancing clinical practice in oral medicine.

#### EVs in oral candidiasis

*C. albicans*, the primary fungal pathogen in the oral cavity, relies heavily on EVs-mediated signalling to enhance its pathogenicity. *C. albicans* EVs contain ***β*-glucans, mannoproteins, proteases, phospholipases**, and metabolic enzymes that contribute to fungal adhesion, hyphal transformation, and epithelial invasion [108].

These vesicles promote:

- Cross-kingdom interactions with bacteria in polymicrobial biofilms
- Immune evasion by modulating macrophage and neutrophil responses
- Oxidative stress and epithelial barrier disruption
- Expansion of biofilm biomass and drug resistance patterns

Fungal EVs therefore represent central molecular vehicles that enable *C. albicans* to thrive in the mucosal environment during infection.

#### EVs in oral squamous cell carcinoma (OSCC)

Since the discovery that cancer cells release large quantities of EVs, research into EVs has exploded, particularly in tumorigenesis due to nature of the products transported by these EVs, their effect on distant cells, and on drug resistance. In oral cancer, tumour-derived EVs promote cancer progression by enhancing cell proliferation, invasion, angiogenesis, and immune suppression [101]. These vesicles carry oncogenic proteins, microRNAs, and matrix-remodelling enzymes that facilitate tumour microenvironment modification and metastasis, while also contributing to resistance to therapy [110]. Tumour-derived EVs promote:

- cell proliferation and invasion.
- epithelial–mesenchymal transition (EMT).
- Induction of angiogenesis through VEGF-related pathways.
- Remodelling of the tumour microenvironment.
- Suppression of anti-tumour immune responses [111,112].

These actions facilitate tumour expansion and increase metastatic potential. Because EVs circulate in saliva and gingival crevicular fluid, tumour-associated EVs molecular signatures can serve as *non-invasive* biomarkers for early OSCC detection, assessment of tumour staging and prognosis [113,114]. Candidate EVs markers include miR-21, miR-31, EGFR, HSP70, and various tumour-associated proteins.

**Figure 4.**
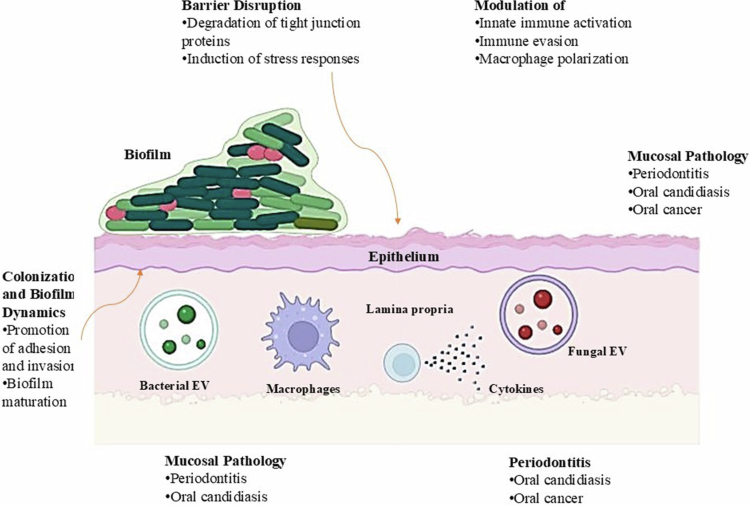
Microbiota-derived extracellular vesicles (EVs) and their interactions with the oral mucosa. This figure illustrates the multifaceted roles of bacterial and fungal extracellular vesicles (EVs) in modulating oral mucosal biology. Microbiota-derived EVs interact with epithelial cells, the lamina propria, and resident immune cells to influence barrier integrity, immune responses, biofilm behaviour, and mucosal disease progression (BioRender Software and ameliorate by chatgpt).

EVs act as central mediators of oral pathology by amplifying inflammation, enhancing microbial virulence, promoting epithelial dysfunction, and driving carcinogenic processes. Host-derived, microbial, and tumour-derived EVs form an intricate network of pathological signals, making EVs both key contributors to disease and promising targets for diagnostic and therapeutic innovation in oral medicine (Figure 4).

## Diagnostic and therapeutic potential of extracellular vesicles in oral medicine

EVs, particularly those present in saliva, have emerged as promising non-invasive biomarkers for the diagnosis and monitoring of both oral and systemic diseases [57]. In oral medicine, their stability in saliva and gingival crevicular fluid, combined with their ability to encapsulate disease-specific molecular cargo, makes them particularly valuable for non-invasive diagnostics, regenerative therapies, and targeted modulation of inflammation and microbial behaviour. This section summarises the main diagnostic and therapeutic implications of EVs in oral health and disease.

EVs function as sensitive indicators of pathological processes due to their ability to carry molecular signatures reflective of their cells of origin. Saliva provides a convenient source of EVs for clinical testing, simplifying sample collection and enabling high patient compliance [108]. Their stability, membrane protection of molecular cargo, and accessibility through simple collection procedures make salivary EVs highly attractive for clinical use [111]. EVs-associated proteins, microRNAs, and DNA fragments have been investigated as diagnostic indicators for oral squamous cell carcinoma (OSCC), periodontal disease, and systemic conditions including diabetes and cardiovascular disease [93,112,115]. For example, specific salivary EVs microRNAs (e.g. miR-21, miR-155) and tumour-derived protein signatures have been associated with OSCC progression and prognosis, supporting their utility in early detection and disease stratification [116]. Similarly, elevated inflammatory and matrix degradation markers in EVs from gingival crevicular fluid correlate with periodontal disease severity, offering potential for disease activity monitoring [117,118].

Beyond diagnostics, EVs hold significant promise as therapeutic agents, particularly in the context of cell-free regenerative medicine [119,120]. EVs derived from dental pulp stem cells and periodontal ligament stem cells contain regenerative microRNAs, growth factors, and immunomodulatory molecules that promote dentin–pulp healing, periodontal regeneration, angiogenesis, and bone formation [91,121]. These properties provide a compelling alternative to stem cell transplantation, offering reduced immunogenicity, improved safety, and easier clinical translation [122]. In addition, EVs are being explored as drug delivery systems due to their natural biocompatibility, ability to cross biological barriers, and inherent targeting capabilities [123]. Their lipid bilayer structures protect therapeutic cargo such as small molecules, nucleic acids, and proteins, allowing controlled release and enhanced delivery to target tissues [124,125]. Engineering strategies, including surface modification and cargo loading, further enhance their potential for targeted therapy in cancer treatment, antimicrobial delivery, and immune modulation [126,127].

The dual role of EVs as diagnostic biomarkers and therapeutic platforms positions them at the forefront of precision oral medicine. Continued advances in EVs isolation, characterisation, and engineering are expected to facilitate their translation into clinical practice, supporting early diagnosis, personalised treatment, and regenerative interventions in oral and systemic diseases.

## Conclusion and future perspectives

EVs have emerged as fundamental mediators of communication within the oral cavity, orchestrating complex interactions among epithelial cells, immune populations, stem cell niches, and the diverse oral microbiota. Their ability to transport bioactive molecules including proteins, lipids, metabolites, and nucleic acids enables EVs to regulate essential physiological processes such as epithelial barrier maintenance, immune homoeostasis, microbial colonisation, and tissue regeneration. Conversely, dysregulation of EVs biogenesis or cargo critically contributes to the onset and progression of major oral diseases, including periodontitis, oral candidiasis, and oral squamous cell carcinoma.

Growing evidence reinforces the dualistic nature of EVs in oral health: while host-derived EVs can promote tissue repair, coordinate immune responses, and maintain microbial balance, microbial EVs often act as potent pathogenic agents, disseminating virulence factors that amplify inflammation, degrade host tissues, and reshape the mucosal microenvironment. Understanding this bidirectional EVs-mediated communication is therefore essential for decoding the mechanisms that link oral dysbiosis, immune dysfunction, and systemic health outcomes [128].

The diagnostic potential of EVs is particularly compelling. Salivary and gingival crevicular fluid EVs carry disease-specific molecular signatures that reflect real-time physiological changes within the oral cavity. These vesicles offer a promising, non-invasive platform for early detection of periodontal disease activity, fungal infections, and oral cancer [57,129]. As high-throughput omics technologies advance, EVs-based biomarker panels may become standard tools in precision oral diagnostics.

Therapeutically, stem-cell-derived EVs represent an emerging class of regenerative agents with the capacity to stimulate dentin–pulp repair, promote periodontal regeneration, and modulate inflammation without the risks associated with whole-cell therapies. Engineering strategies to load EVs with specific microRNAs, proteins, or small molecules hold great promise for developing next-generation immunomodulatory and antimicrobial interventions. Additionally, targeting microbial EVs pathways by neutralising virulence factor–rich OMVs or inhibiting their biogenesis could open new avenues for managing dysbiosis-driven diseases.

Despite these advances, several challenges must be addressed before EVs-based applications are fully integrated into clinical practice. Current obstacles include the lack of standardised EV isolation and characterisation protocols, difficulty differentiating between host- and microbe-derived vesicles in heterogeneous samples and limited in vivo understanding of EV biodistribution and functional dynamics within the oral cavity. Rigorous clinical studies, regulatory frameworks, and scalable EV manufacturing platforms will be essential to overcome these barriers.

## Conclusion

EVs play a central and multifaceted role in shaping oral health and disease. Their diagnostic and therapeutic potential offers exciting opportunities for the future of oral medicine, particularly in the field of regenerative dentistry, precision immunotherapy, and early cancer detection. Continued interdisciplinary research integrating microbiology, immunology, nanotechnology, and clinical sciences will be key to unlocking the full translational impact of extracellular vesicles in oral health care.
